# Overestimation of Survival Rates of Cardiopulmonary Resuscitation Is Associated with Higher Preferences to Be Resuscitated: Evidence from a National Survey of Older Adults in Switzerland

**DOI:** 10.1177/0272989X231218691

**Published:** 2023-12-29

**Authors:** Clément Meier, Sarah Vilpert, Maud Wieczorek, Gian Domenico Borasio, Ralf J. Jox, Jürgen Maurer

**Affiliations:** Faculty of Biology and Medicine and the Faculty of Business and Economics, University of Lausanne, Switzerland; Swiss Centre of Expertise in the Social Sciences, Lausanne, Switzerland; Swiss Centre of Expertise in the Social Sciences, Lausanne, Switzerland; Faculty of Business and Economics, University of Lausanne, Switzerland; Swiss National Centre of Competence in Research LIVES – Overcoming Vulnerability: Life Course Perspectives, Lausanne and Geneva, Switzerland; Palliative and Supportive Care Service, Lausanne University Hospital and University of Lausanne, Lausanne, Switzerland; Palliative and Supportive Care Service, Chair in Geriatric Palliative Care, and Institute of Humanities in Medicine, Lausanne University Hospital and University of Lausanne, Switzerland; Faculty of Business and Economics, University of Lausanne, Switzerland

**Keywords:** CPR survival rates, advance directives, cardiac arrest, end-of-life preferences, end-of-life care decisions

## Abstract

**Background:**

Many widely used advance directives templates include direct questions on individuals’ preferences for cardiopulmonary resuscitation (CPR) in case of decision-making incapacity during medical emergencies. However, as knowledge of the survival rates of CPR is often limited, individuals’ advance decisions on CPR may be poorly aligned with their preferences if false beliefs about the survival rates of CPR shape stated preferences for CPR.

**Methods:**

We analyzed nationally representative data from 1,469 adults aged 58+ y who responded to wave 8 (2019/2020) of the Swiss version of the Survey on Health, Ageing, and Retirement in Europe (SHARE) to assess the partial association between knowledge of CPR survival rates and stated preferences for CPR using multivariable probit regression models that adjust for social, health, and regional characteristics. Knowledge of CPR survival rates was assessed by asking how likely it is in general in Switzerland for a 70-y-old to survive until hospital discharge from a CPR performed outside of a hospital. Preferences for CPR were measured by asking respondents if they would wish to be resuscitated in case of cardiac arrest.

**Results:**

Only 9.3% of respondents correctly assessed the chances for a 70-y-old to survive until hospital discharge from a CPR performed outside of a hospital, while 65.2% indicated a preference to be resuscitated in case of a cardiac arrest. Respondents who correctly assessed CPR survival were significantly more likely to wish not to be resuscitated (average marginal effect: 0.18, *P* < 0.001).

**Conclusions:**

Reducing misconceptions concerning the survival rates of CPR could change older adults’ preferences for CPR and make them more likely to forgo such treatments.

**Highlights:**

- Many older adults in Switzerland overestimate the survival rates of cardiopulmonary resuscitation (CPR).
- The study reveals that individuals with accurate knowledge of CPR survival rates are more likely to refuse resuscitation in case of cardiac arrest.
- Overestimation of CPR survival rates may lead to a mismatch between individuals’ preferences for CPR and their actual end-of-life care decisions.
- Improving the general population’s knowledge of CPR survival rates is crucial to ensure informed decision making and effective advance care planning.

Increasing life spans and improvements in medical technologies have led to major changes in the social and medical contexts in which death occurs.^1,2^ The end of life and death are now more predictable, and as a result, it is increasingly important for individuals to communicate their preferences for end-of-life care in case they become unable to make decisions themselves.^3,4^ Decisions regarding life-supporting treatment in advance directive forms commonly include questions on preferences for cardiopulmonary resuscitation (CPR), asking individuals to indicate whether they would like to be resuscitated in case of cardiac arrest.^5,6^ In Switzerland, the survival rate for a 70-y-old after a CPR outside of a hospital is lower than 8%.^7,8^ However, CPR survival rates are often overestimated in the general population,^9,10^ which might skew individuals’ decisions toward CPR and result in poor alignment between their advance directives and actual end-of-life care preferences,^11^ especially as many people appear to be relatively critical with regard to potential “overtreatment” in case of medical emergencies.^12,13^ Previous studies focusing on patients showed that those who received correct information on actual CPR survival rates were more likely to refuse to be resuscitated in case of a cardiac arrest.^14–17^ Despite a few studies that describe the lack of knowledge of CPR survival rate and its potential association with patients’ preferences for CPR,^14–17^ little is known about this association outside of the clinical settings. To fill this knowledge gap, we used nationally representative data on adults aged 58 y and older in Switzerland to explore the association between preferences to be resuscitated in case of cardiac arrest and knowledge of CPR survival rates.

## Methods

### Study Design and Participants

We used data of individuals who answered a Switzerland-specific self-administered paper-and-pencil questionnaire as part of wave 8 of the Survey on Health, Ageing, and Retirement in Europe (SHARE).^18^ Data collection took place between October 2019 and the beginning of March 2020. In total, 2,005 target respondents and their partners participated in the in-person interview of SHARE wave 8 in Switzerland, of which 1,891 (94,3%) also completed the national paper-and-pencil questionnaire. As the SHARE sample of individuals aged 50 y and older in Switzerland was last refreshed in 2011, our current sample does not include target individuals aged 50 to 57 y anymore. We therefore focus only on respondents aged 58 y and older and drop partners of target respondents below age 58 y. After also excluding respondents with missing responses on variables used in the analysis, the final number of respondents in our sample consisted of 1,469.

### Outcome Variable

#### Preference for CPR

Respondents’ preference for CPR was assessed based on a hypothetical question (Appendix 1): *Imagine that you experience a cardiac and/or respiratory arrest. In this situation, do you wish to be resuscitated or not to be resuscitated (0* *=* *refuse CPR, 1* *=* *accept CPR)*?

### Exposure

#### Respondents’ knowledge of CPR survival rate

The questionnaire included a vignette that asked respondents to estimate how likely it is for a 70-y-old citizen in Switzerland to survive until hospital discharge after an out-of-hospital CPR following a cardiac arrest (Appendix 1). The participant could choose among 4 possible answers (very unlikely [0–25%], rather unlikely [26–50%], rather likely [51–75%], and very likely [76–100%]).

### Covariates

Building on a seminal work investigating the end-of-life medical preferences of older adults in Switzerland,^19^ our statistical models include information on sex (male, female), age (58–64 y, 65–74 y, 75+ y), education levels (low = International Standard Classification of Education [ISCED] levels 0, 1, and 2; middle = ISCED levels 3–4; high = ISCED levels 5–6),^20^ partnership status (has a partner, has no partner), subjective financial difficulties (ability to make ends meet: easily, fairly easily, and with difficulty), Switzerland’s linguistic regions (German, French, or Italian), living area (urban, rural), self-rated health (poor/fair health, good health, very good/excellent health), and activities of daily living limitations (no, yes).

### Statistical Analysis

The distribution of preferences for CPR and knowledge of CPR survival rates were calculated using weighted proportion estimation with corresponding 95% confidence intervals. To calibrate the sample and obtain estimates representative of the target population of older adults aged 58 y and older, we used the cross-sectional weights available in the SHARE data set.^18^ The partial associations between preferences for CPR and knowledge of CPR survival rates were assessed using unweighted multivariable probit regression models adjusting for the above-mentioned individuals’ social, health, and regional characteristics. Two separate probit regression models were run, one with knowledge of CPR survival rates as a binary variable (right answer, wrong answer) and the other with the 4 possible answer variables (very unlikely [0–25%], rather unlikely [26–50%], rather likely [51–75%], and very likely [76–100%]). All estimations used STATA/SE 17.0 software (STATA Corporation, College Station, TX), and results were reported as average marginal effects (AME) along with corresponding standard errors (SE) clustered at the household level.

## Results

**Figure 1:**
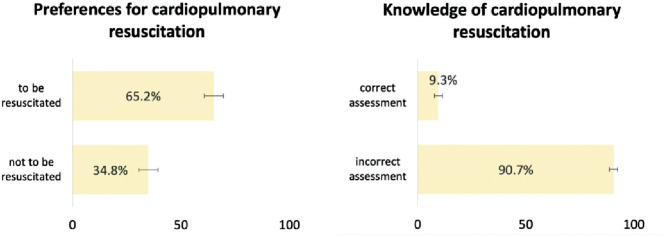
Preferences for cardiopulmonary resuscitation and knowledge of survival rates, weighted proportions, and 95% confidence intervals, adults aged 58+ y, SHARE Switzerland, 2019/2020, *N* = 1,469. The figures present the weighted distribution of preferences for cardiopulmonary resuscitation and knowledge of survival rates.

The characteristics of the study population are presented in Appendix 2. Figure 1 shows the weighted distribution of preference for CPR and knowledge of survival rates. In our sample, 65.2% of respondents wished to be resuscitated in case of a cardiac arrest, while only 9.3% of respondents correctly stated that it is very unlikely (0–25%) in general in Switzerland for a 70-y-old to survive until hospital discharge from a CPR performed outside of a hospital following a cardiac arrest.

The partial associations of preference for CPR with knowledge of CPR survival rates are presented in Table 1, adjusting for social, regional, and health factors. Additional information about the associations between the covariates and the outcome variables can be found in Appendix 3. Respondents who correctly estimated the CPR survival rate were significantly more likely not to want to be resuscitated in case of a cardiac and/or respiratory arrest (AME: 0.18, *P* < 0.001). In addition, when regressing preferences for CPR on stated beliefs regarding CPR survival as measured by all 4 possible categories, respondents who reported that CPR survival would be “rather likely” (51–75%) and “very likely” (76–100%) were significantly less likely to refuse CPR for themselves (AME: −0.23, *P* < 0.001 and AME: −0.22, *P* < 0.001). The 2 probit analyses conducted to validate the robustness of our findings revealed consistent outcomes across both models. In each model, individuals who correctly assessed the likelihood of surviving CPR were more inclined to opt against resuscitation. Furthermore, the second probit regression indicated that the association was statistically significant only for individuals who estimated the CPR survival rate to be higher than 50%.

**Table 1:** Partial Associations of Preference for Cardiopulmonary Resuscitation on Knowledge of Survival Rates Controlling for Respondents’ Social, Cultural, and Health Characteristics, Adults Aged 58+ y, SHARE Switzerland, 2019/2020, *N* = 1,469^a^

	Preference Not to Be Resuscitated (AME)	Preference Not to Be Resuscitated (AME)
**Gave the correct assessment** (gave the incorrect assessment)	0.18*** (0.04)	
**Likelihood of surviving from cardiopulmonary resuscitation** (very unlikely [0–25%])		
Rather unlikely (26–50%)		−0.08 (0.04)
Rather likely (51–75%)		−0.23*** (0.04)
Very likely (76–100%)		−0.22*** (0.05)

AME, average marginal effects.

a The table shows average marginal effects and standard errors in parentheses from separate models. The 2 probit regression models control for sex, age, education levels, partnership status, subjective financial situation, linguistic region, living area, self-rated health, and limitations on activities of daily living.

*** *P* < 0.001.

## Discussion

Using a population-based sample of 1,469 adults aged 58 y and older living in Switzerland, we investigated the association between preferences to be resuscitated in case of cardiac arrest and knowledge of general CPR survival rates assessed via a survey vignette. Most respondents (65.2%) wished to be resuscitated in case of cardiac arrest and overestimated (90.7%) the probability for a 70-y-old to survive until hospital discharge from a CPR performed outside of a hospital. In addition, respondents who overestimated CPR survival rates were statistically significantly more likely to want to be resuscitated in case of cardiac arrest.

Only 9.3% had reasonably accurate perceptions of these CPR survival rates. More accurate perceptions of CPR survival rates are positively associated with refusal of CPR, consistent with the results of a comparable study on older patients in a clinical setting.^17^ More accurate perceptions of CPR survival rates, which are much lower than usually perceived, could influence older adults’ preference for CPR and corresponding choices in potential advance directives. Similar findings have also been obtained in earlier intervention studies^14–16^ that directly provided patients with information about the probability of CPR survival before asking for their preferences. In these studies, most respondents did not want to undergo CPR once health care providers explained to them the probability of survival.

Unrealistic expectations of CPR survival may also require health care providers to manage the disappointment of patients and their families when discussing actual chances of survival following CPR.^21^ Health care providers often report the struggle to initiate CPR discussions knowing that the topic is emotionally difficult for patients and depends on patients’ evolution of their prognoses, which is hard to predict.^22^ Improving individuals’ knowledge of CPR survival rates could facilitate discussions with health care providers and potentially affect treatment preferences by mitigating unrealistic expectations.^23^ Moreover, the fact that most people overestimate the chances of survival of CPR strongly supports an advance care planning process that would include medical expertise on prognosis, for instance, in the form of CPR decision aids.^24^

### Limitations

A primary limitation of our study is the phrasing of the resuscitation question, which did not provide alternatives such as comfort measures. This might have inclined participants toward CPR, potentially perceiving it as the only option against abandonment, thereby potentially overestimating the overall preference for resuscitation. Furthermore, using qualitative and quantitative labels for the answer categories in the question on knowledge of CPR survival rates could have confused some respondents. In addition, the concepts of probability may be hard to understand for some respondents, even though probabilistic questions have been successfully used in SHARE for quite some time. Similarly, problems of study enrollment, attrition, and missing data may create a sample selection bias and underrepresentation of especially vulnerable segments of the population.

## Conclusion

Most older adults in Switzerland wish to be resuscitated in case of cardiac arrest, but those with more accurate beliefs regarding CPR survival rates are more likely to prefer not to be resuscitated in the case of a cardiac arrest. The overestimations of CPR survival rate may result in patients and families choosing to be resuscitated in case of a cardiac arrest due to overoptimistic beliefs regarding the likelihood of surviving CPR, which could compromise the quality and reliability of advance care planning. Therefore, it is necessary to improve the general population’s knowledge of the CPR survival rate and inform the health care providers of the importance of discussing this treatment and its consequences in detail with their patients.

## Supplemental Material

sj-docx-1-mdm-10.1177_0272989X231218691 – Supplemental material for Overestimation of Survival Rates of Cardiopulmonary Resuscitation Is Associated with Higher Preferences to Be Resuscitated: Evidence from a National Survey of Older Adults in Switzerland Supplemental material, sj-docx-1-mdm-10.1177_0272989X231218691 for Overestimation of Survival Rates of Cardiopulmonary Resuscitation Is Associated with Higher Preferences to Be Resuscitated: Evidence from a National Survey of Older Adults in Switzerland by Clément Meier, Sarah Vilpert, Maud Wieczorek, Gian Domenico Borasio, Ralf J. Jox and Jürgen Maurer in Medical Decision Making
