# Supplementary material for: Overestimation of Survival Rates of Cardiopulmonary Resuscitation Is Associated with Higher Preferences to Be Resuscitated: Evidence from a National Survey of Older Adults in Switzerland
Source: Med Decis Making. 2023 Dec 29;44(2):129–34. doi: 10.1177/0272989X231218691 (PMC10865767; doi:10.1177/0272989X231218691)
Supplement: sj-docx-1-mdm-10.1177_0272989X231218691 – Supplemental material for Overestimation of Survival Rates of Cardiopulmonary Resuscitation Is Associated with Higher Preferences to Be Resuscitated: Evidence from a National Survey of Older Adults in Switzerland [file sj-docx-1-mdm-10.1177_0272989X231218691.docx]

**Appendices**

| **Appendix 1: CPR questions used in the analysis** |
| --- |
| **Question 1:** Imagine that you experience a cardiac and/or respiratory arrest. In this situation, you wish ...  Answer categories: "**to be** resuscitated", " **not to be** resuscitated"  **Question 2:** People have representations of end-of-life medical situations. We would like to know yours. We would like to know whether you think that the situations described below and related to end-of-life are very unlikely (0-25%), rather unlikely (26-50%), rather likely (51-75%) or very likely (76-100%). **If you don’t know, please give us your best estimate.** *Example: “In your opinion, what are the chances that it is snowing tomorrow?” If you tick “very likely”, you consider that the chances that it is snowing tomorrow*  *range between 76% and 100%.*  In your opinion, how likely is it **in general in Switzerland** for a 70-year-old to survive until hospital discharge from a cardiopulmonary resuscitation performed outside of a hospital following a cardiac arrest?  Answer categories: "very unlikely (0-25%)", "rather unlikely (26-50%)", "rather likely (51-75%)", "very likely (76-100%)" |

| **Appendix 2: Characteristics of the study population, adults aged 58+, SHARE Switzerland, 2019/2020, n=1,469** | | | |
| --- | --- | --- | --- |
|  | Unweighted | Weighted | |
|  | n | % | CI |
| **Sex** |  |  |  |
| male | 699 | 53.0 | [48.5-57.4] |
| female | 770 | 47.0 | [42.6-51.5] |
| **Age groups** |  |  |  |
| 58-64 years | 370 | 50.9 | [45.7-56.0] |
| 65-74 years | 631 | 27.9 | [24.7-31.4] |
| 75+ years | 468 | 21.2 | [18.6-24.1] |
| **Education** |  |  |  |
| low | 237 | 14.2 | [11.4-17.7] |
| middle | 944 | 64.9 | [60.1-69.5] |
| high | 288 | 20.9 | [16.9-25.4] |
| **Partnership status** |  |  |  |
| has a partner | 1,112 | 70.7 | [65.8-75.1] |
| no partner | 357 | 29.3 | [24.9-34.2] |
| **Make ends meet** |  |  |  |
| easily | 817 | 57.7 | [52.8-62.4] |
| fairly easily | 468 | 30.2 | [26.0-34.8] |
| with difficulty | 184 | 12.1 | [9.3-15.6] |
| **Language** |  |  |  |
| German | 1,064 | 73.0 | [68.1-77.4] |
| French | 352 | 24.2 | [19.8-29.0] |
| Italian | 53 | 2.9 | [2.0-4.2] |
| **Living area** |  |  |  |
| urban | 666 | 40.9 | [36.1-45.9] |
| rural | 803 | 59.1 | [54.1-63.9] |
| **Self-rated health** |  |  |  |
| poor/fair health | 266 | 16.1 | [13.0-19.7] |
| good health | 617 | 39.5 | [34.8-44.4] |
| Very good/excellent health | 586 | 44.4 | [39.3-49.7] |
| **ADL limitations** |  |  |  |
| no | 1,378 | 94.2 | [91.7-96.0] |
| yes | 91 | 5.8 | [4.0-8.3] |
| **Preferences for CPR** |  |  |  |
| to be resuscitated | 873 | 65.2 | [60.7-69.4] |
| not to be resuscitated | 596 | 34.8 | [30.6-39.3] |
| **Knowledge of CPR** |  |  |  |
| incorrect assessment | 1,288 | 90.7 | [88.7-92.3] |
| correct assessment | 181 | 9.3 | [7.7-11.3] |
| *Note, unweighted and weighted number of observations for the whole sample. n = number; CI = confidence interval; ADL = activities of daily living limitations.* | | | |

| **Appendix 3: Partial associations of preference for cardiopulmonary resuscitation on knowledge of survival rates controlling for respondents’ social, cultural, and health characteristics, adults aged 58+, SHARE Switzerland, 2019/2020, n=1,469** | | |
| --- | --- | --- |
|  | Preference not to be resuscitated  (AME) | Preference not to be resuscitated  (AME) |
| **Gave the correct assessment** (gave the incorrect assessment) | 0.18^***^  (0.04) |  |
| **Likelihood of surviving from cardiopulmonary resuscitation** (Very unlikely (0-25%)) |  |  |
| Rather unlikely (26-50%) |  | -0.08 |
|  |  | (0.04) |
| Rather likely (51-75%) |  | -0.23^***^ |
|  |  | (0.04) |
| Very likely (76-100%) |  | -0.22^***^ |
|  |  | (0.05) |
| **Sex (male)** |  |  |
| female | 0.11^***^ | 0.11^***^ |
|  | (0.02) | (0.02) |
| **Age groups** (58-64 years) |  |  |
| 65-74 years | 0.10^**^ | 0.10^**^ |
|  | (0.03) | (0.03) |
| 75+ years | 0.19^***^ | 0.19^***^ |
|  | (0.03) | (0.03) |
| **Education** (low) |  |  |
| middle | -0.02 | -0.01 |
|  | (0.04) | (0.03) |
| high | -0.11^*^ | -0.10^*^ |
|  | (0.04) | (0.04) |
| **Partnership status** (has a partner) |  |  |
| no partner | 0.08^*^ | 0.07^*^ |
|  | (0.03) | (0.03) |
| **Make ends meet** (easily) |  |  |
| fairly easily | -0.02 | -0.02 |
|  | (0.03) | (0.03) |
| with difficulty | -0.05 | -0.05 |
|  | (0.04) | (0.04) |
| **Language** (German) |  |  |
| French | 0.07^*^ | 0.08^**^ |
|  | (0.03) | (0.03) |
| Italian | -0.26^***^ | -0.26^***^ |
|  | (0.04) | (0.04) |
| **Living area** (urban) |  |  |
| rural | -0.01 | -0.01 |
|  | (0.03) | (0.03) |
| **Self-rated health** (poor/fair health) |  |  |
| good health | -0.10^**^ | -0.09^*^ |
|  | (0.04) | (0.04) |
| very good/excellent health | -0.12^**^ | -0.11^**^ |
|  | (0.04) | (0.04) |
| **ADL limitations** (no) |  |  |
| yes | 0.02 | 0.01 |
|  | (0.05) | (0.05) |
| Observations | 1469 | 1469 |
| *The table shows average marginal effects and standard errors in parentheses from separate models. Statistical significance: * p < 0.05, ** p < 0.01, *** p < 0.001.* | | |
